# Bone Changes in the Temporomandibular Joint: A Retrospective Cone Beam Computed Tomography Study

**DOI:** 10.3390/dj14050313

**Published:** 2026-05-20

**Authors:** Daniela Pereira Urgal, Carolina Oliveira de Lima, João Victor Frazão Câmara, Isabel Ferreira Barbosa, Maira do Prado, Celso Neiva Campos

**Affiliations:** 1 Department of Dental Clinic, School of Dentistry, Federal University of Juiz de Fora, Campus Universitário, Juiz de Fora 36036-330, MG, Brazil; danielaurgal1@gmail.com (D.P.U.); c.oliveiradelima@ufjf.br (C.O.d.L.); cncampos@terra.com.br (C.N.C.); 2 Clinic of Operative Dentistry, Periodontology and Preventive Dentistry, Saarland University, 66424 Homburg, Saar, Germany; 3 São Leopoldo Mandic Institute, Rio de Janeiro 22221-070, RJ, Brazil; barbosa.isabelferreira@gmail.com; 4 School of Dentistry, Veiga de Almeida University, Rio de Janeiro 20271-901, RJ, Brazil; mairapr@hotmail.com

**Keywords:** cone-beam computed tomography, degenerative temporomandibular joint disease, temporomandibular joint morphology

## Abstract

**Objectives:** To analyze the prevalence of bone changes in the temporomandibular joint (TMJ) and describe their distribution regarding age and sex using Cone Beam Computed Tomography (CBCT). **Methods:** This retrospective study analyzed CBCT images of 483 individuals (326 females, 157 males) retrieved from a computer database and assessed using the iCAT Workstation. Right and left condyles were evaluated for the presence of flattening, osteophytes, sclerosis, erosion, and subchondral cysts. Pearson’s chi-square test was used to identify potential associations between these alterations and demographic variables (*p* < 0.05). **Results:** At least one TMJ alteration was observed in 91.5% of the participants. Flattening was the most frequent finding (76.4%), followed by osteophytes (53%), sclerosis (32.3%), erosion (20.1%), and subchondral cysts (2.7%). No statistically significant association was found between gender and the presence of alterations (*p* > 0.05), with a high prevalence in both females (91.1%) and males (92.4%). However, specific degenerative changes showed a significant upward trend with age: osteophytes increased from 43.9% in patients under 20 to 68.9% in those over 60, while erosion doubled from 12.2% to 24.4% in the same groups (*p* < 0.05). Alterations were slightly more frequent on the left side (81.6%) than on the right side (76.6%). **Conclusions:** Degenerative TMJ changes are highly prevalent in the studied population, with flattening appearing as a widespread finding across all groups. While some specific alterations, such as osteophytes and erosion, show an increased prevalence in older age groups, these associations reflect a descriptive trend of bone remodeling over time rather than a direct causal relationship.

## 1. Introduction

The temporomandibular joint (TMJ) is a complex joint in the human body that is related to chewing, speaking and swallowing movements [1]. In order to maintain homeostasis in the face of the continuous forces that the stomatognathic system is subjected to, processes of tissue adaptation and bone remodeling take place, which is considered a physiological process aimed at adapting the structure of the TMJ to the mechanical forces applied to the joint. It is an essential biological response to normal functional demands, ensuring joint homeostasis and occlusal function [2,3]. However, when these forces exceed the individual’s adaptive capacity, painful processes called Temporomandibular Dysfunction (TMD) begin.

TMD affects muscles, joints and other surrounding anatomical structures, creating conditions that are difficult to diagnose [2,3]. Due to the complexity of the structures involved in full TMJ function, a detailed history and clinical examination are essential to locate the source of the dysfunction and to establish the differential diagnosis of patients with TMJ [4,5]. Moreover, postural, psychosomatic, psychological factors, other morbidities and factors related to the anatomy of the TMJ, such as bone, neuromuscular, dental and joint components may be involved [6,7]. It is important to have a depth knowledge of the different types of bone changes of TMJ and its images characteristics. In musculoskeletal imaging, several structural changes are commonly described to characterize joint pathology. Flattening refers to the loss of the normal convex contour of an articular surface, typically resulting from chronic mechanical stress and degeneration [6,7]. Osteophytes are marginal bony outgrowths that develop at joint edges as part of a reparative response to cartilage loss and altered joint mechanics, frequently observed in degenerative joint disease. Sclerosis denotes an increase in subchondral bone density, appearing as areas of increased radiopacity on imaging, and reflects adaptive bone remodeling in response to increased load transmission [6,7]. In contrast, erosion describes focal loss of bone at the articular surface, often associated with inflammatory processes that lead to structural damage. Finally, subchondral cysts are well-defined, fluid-filled cavities located within the subchondral bone, believed to arise from synovial fluid intrusion or bone necrosis secondary to chronic stress. Collectively, these features provide important insights into the underlying pathophysiological mechanisms of joint degeneration and disease progression [6,7].

Among the imaging tests for the TMJ, planography is widely used, however, due to the overlapping of anatomical structures and low level of detail, sometimes another radiographic method is necessary for a complete evaluation. Cone beam computed tomography (CBCT), on the other hand, allows the anatomy of the TMJ to be examined without overlapping and distortion of bone morphology, joint space, and assessment of dynamic function in all three dimensions and without distortions [8,9], being a useful method in the most diverse specialties for obtaining a diagnosis in the face of pathologies and in the development of therapeutic planning [10,11].

Although prevalence studies are often considered descriptive, assessing the frequency and pattern of bone changes in the temporomandibular joint (TMJ) through CBCT imaging has important clinical implications. Knowledge of the prevalence and distribution of these alterations contributes to improved diagnostic accuracy, supports clinical decision-making, and helps distinguish between adaptive remodeling and pathological conditions. Furthermore, establishing baseline prevalence data is essential for future studies aiming to correlate imaging findings with clinical symptoms and functional impairment, thereby enhancing the understanding of TMJ disorders in different populations.

Thus, the aim of this study was to analyze the prevalence of alterations that characterize bone degenerations in the TMJ, based on cone beam computed tomography images. The null hypothesis to be tested is that there is no relation between temporomandibular joint changes and (I) the age (II) and gender of the patients.

## 2. Materials and Methods

### 2.1. Ethical Aspects

The research was ethically developed according to the Declaration of Helsinki, and it was approved by the Ethics and Research Committee of the Federal University of Juiz de Fora under number 3.123.671 at 28.01.2019. Prior to ethical approval, only a preliminary feasibility assessment and identification of potentially eligible records were performed. No data extraction, image measurements, statistical analyses, study-specific consent procedures, or any other research-related activities were conducted before ethics approval was granted. All CBCT scans included in this study had been previously acquired exclusively for clinical and diagnostic purposes as part of routine patient care. No CBCT scan was performed specifically for this research, and participants were not exposed to any additional radiation related to the study. After ethics approval, all patient data and CBCT records were anonymized/de-identified prior to analysis to ensure confidentiality and privacy protection. The anonymization process removed all personally identifiable information before data evaluation. The requirement for informed consent was waived by the Ethics Committee due to the retrospective nature of the study and the use of anonymized data.

### 2.2. Study Characterization

This is a cross-sectional observational study investigating the prevalence of alterations in the Temporomandibular Joint (TMJ) detected in cone beam computed tomography images.

### 2.3. Study Sample

**Figure 1:**
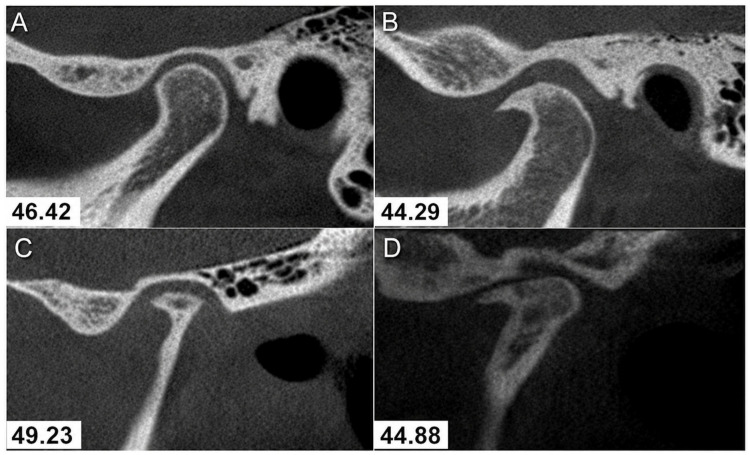
Condyle assessment: (**A**) normal condition; (**B**) osteophyte and sclerosis; (**C**) flattening, osteophyte, sclerosis, and subchondral cyst; (**D**) flattening, osteophyte, sclerosis, and erosion.

The images of 500 patients were examined, totaling 1.000 TMJ analyzed (*n* = 1.000), obtained from the database of the Radiology Department of the School of Dentistry of the Federal University of Juiz de Fora. This study sought to identify the presence of alterations such as flattening, osteophytes, sclerosis, erosion and subchondral cysts (Figure 1) and to correlate the presence of these alterations with the age and sex of the patients.

The exams with a bone fixation plate suggestive of postoperative trauma to the mandible, exams of individuals who had no posterior teeth or who had all their posterior teeth treated endodontically were excluded from the sample (17 images of patients). Thus, 483 images of patients were included.

### 2.4. Data Source

The images were acquired by the same CT scanner (I-Cat^®^, Imaging Sciences International, Hatfield, PA, USA), with the following acquisition protocol: 120 kV, 3–8 mA, 26.9 s rotation time, 8 cm field of view, 0.25 mm slice. The examination was carried out with the patients seated, using the median sagittal plane perpendicular to the ground and the occlusal plane parallel to the ground as positioning references. A headband and chin positioner were used to prevent the patient’s head from moving. To ensure methodological consistency and minimize confounding variables, only CBCT scans from the database that were acquired using the exact same exposure and acquisition protocol were added. The pathologies were qualitatively assessed based on the study of Shahidi et al., 2018 [12].

Initially, using the ICatVision software (version 1.8.1.10) on the “TMJ” viewing screen, the condyle was centered from the axial section. For a more detailed assessment, 0.25 mm thick sections were used. The images were first evaluated using the axial section, followed by the paracoronal, sagittal and parasagittal sections. The images were evaluated by an undergraduate student who underwent calibration with specialist dentists and doctors in dental radiology and the surface of the condyle and eminence bone is classified according to the methodology by the presence or health of flattening, erosion, osteophyte, sclerosis and subchondral cyst [12].

These conditions can appear alone or in combination. As for the radiographic criterion of flattening, the loss of convexity or concavity of the common contours is observed. The local growth of bones arising from a mineralized articular surface can be characterized as osteophyte. Sclerosis is an area of increased density of cortical bone that extends into the bone marrow and subchondral cyst is a small circular hyperdense area with regular margins surrounded by variable areas of increased density. Erosion is an area of decreased density of the cortical bone and adjacent subcortical bone [13]. Calibration was carried out by presenting clinical cases. After calibration, the Kappa test was developed, in which the student evaluated 10% of the sample at two different times to assess the reliability of the diagnostic criteria. The right and left condyles were analyzed separately, and the alterations of interest were flattening, osteophyte, sclerosis, erosion and subchondral cyst.

### 2.5. Data Analysis

Records of each patient like age, sex and condylar changes in both sides of dental arch were added in a table in the Excel program version 2010. The presence of each degeneration was given different names, with flattening, osteophyte, sclerosis, erosion and subchondral cyst being represented by numbers 1, 2, 3, 4 and 5, respectively. The absence of condylar change was considered when the condyles had a smooth, clear cortical bone surface and it was represented by the number 0 [14].

### 2.6. Statistical Analysis

The data was statistically analyzed by IBM SPSS Statistics software (version 15.0; IBM Corp, Armonk, NY, USA) using Pearson’s chi-square test to check for the existence of relationship between the presence of different TMJ alterations with gender, age and side of the dental arch. In addition to the chi-square test, effect size was estimated using Cramér’s V to quantify the strength of the associations. The level of statistical significance was set 5% (*p* < 0.05).

## 3. Results

The images of 483 patients were assessed in this study (326 women and 157 men). The frequencies of TMJ flattening, osteophytes, sclerosis, erosion and subchondral cysts in relation to gender are shown in Table 1. The presence of at least one TMJ alteration was observed in 442 patients (91.5%) of the patients assessed. Flattening was the most common alteration found in this study (76.4%), followed by osteophyte (53%), sclerosis (32.3%), erosion (20.1%) and subchondral cyst (2.7%). Regarding gender, the presence of TMJ alterations showed no statistical difference in the right TMJ ($\chi^{2}=0.315$, p=0.575, Cramér’s V = 0.026) or the left TMJ ($\chi^{2}=0.384$, p=0.535, Cramér’s V = 0.028), with an overall prevalence of 91.1% in females and 92.4% in males (Table 1). These results indicate that degenerative changes affect both sexes almost equally in the studied population.

Regarding age, a statistically significant relationship was observed between advancing age and the presence of osteophytes, sclerosis, erosion, and subchondral cysts (*p* < 0.05, Table 2). The prevalence of osteophytes increased progressively with age, rising from 43.9% in patients under 20 years old to 68.9% in those aged 60 or older (*p* < 0.05). Sclerosis was found in 24.4% of the youngest group, reaching 44.4% in the group aged 60 or older (*p* < 0.05, Cramér’s V = 0.122). Similarly, erosion presented a prevalence of 12.2% in younger patients, which effectively doubled to 24.4% in patients aged 60 and over (*p* < 0.05, Cramér’s V = 0.141). Regarding subchondral cysts, no cases were detected in patients under 20 years of age, whereas a prevalence of 6.7% was observed in the group aged 60 or older (*p* < 0.05, Cramér’s V = 0.123). Unlike the other degenerative changes, flattening did not show a statistically significant association with age (*p* > 0.05), maintaining a high prevalence across all groups, such as 78% to 82.2%.

The analysis of laterality showed that the left TMJ was more frequently affected than the right TMJ for overall degenerative changes (81.6% vs. 76.6%; McNemar’s test, $\chi^{2}=4.408$, p=0.036, effect size = 0.050), indicating a small but statistically significant asymmetry between sides. Specifically, prevalence rates for the left and right sides, respectively, were flattening (62.3% vs. 56.3%; $\chi^{2}=4.556$, p=0.033, effect size = 0.058), osteophytes (38.9% vs. 36.4%; $\chi^{2}=0.176$, p=0.675, effect size = 0.012), sclerosis (25.3% vs. 17.6%; $\chi^{2}=14.679$, p<0.001, effect size = 0.085), erosion (12.2% vs. 11.6%; $\chi^{2}=0.000$, p=1.000, effect size = 0.002), and subchondral cysts (1.2% vs. 1.4%; $\chi^{2}=0.000$, p=1.000, effect size = 0.002). These findings indicate that the statistically significant laterality differences were driven mainly by flattening and sclerosis, although the magnitude of the effect was small (Table 3).

## 4. Discussion

The temporomandibular joint (TMJ) often presents bone degeneration such as erosion, subchondral cyst, sclerosis and osteophytes [14] which can be challenging to detect on conventional radiography due to the overlapping of anatomical structures [15,16].

Several studies have evaluated TMJ bone changes using magnetic resonance imaging (MRI) [11,17,18,19], which is considered the gold standard for studying TMJ soft tissues. However, the exam has little ability to show articular bone changes, especially related to the condylar and temporal cortices. Conventional tomography (CT) has also been studied, with digital CT being a promising technique due to its low cost and low radiation dose [17]. In the present study, the CBCT was used as a diagnostic tool to establish the prevalence of the morphological changes of the mandibular condyle, such as flattening, osteophytes, sclerosis, erosion and subchondral cysts, as this technique is considered the gold standard for TMJ bone analysis [14,16,19,20,21,22,23,24]. According to an anterior study, CBCT evaluation results in modification of the primary diagnosis after clinical examination in 26.08% of cases [19].

According to our results, there was a high prevalence (91.5%) of at least one TMJ alteration in the patients assessed, a fact that corroborates with previous studies that demonstrated prevalence of TMJ alterations greater than 90% [12,13]. However, it is important to consider that these studies may differ substantially in terms of study design, sample characteristics, and inclusion criteria, which can directly influence prevalence estimates. For example, variations in age distribution, presence of symptomatic versus asymptomatic individuals, and imaging protocols may account for similarities or discrepancies across studies, limiting direct comparability. Considering total values, flattening was the most frequently found alteration in this study (76.4%), followed by osteophyte (53%), sclerosis (32.3%), erosion (20.1%) and subchondral cyst (2.7%). This result corroborates the anterior studies that also found the flattening as the most frequently alteration followed by irregularities and osteophytes [12,14]. Notably, differences in diagnostic criteria and imaging resolution across studies may influence the detection rate of subtle changes such as flattening, which is often interpreted variably as either physiological remodeling or early degenerative change.

It is important to mention that the exclusion of individuals with postoperative mandibular trauma, absence of posterior teeth, or complete endodontic treatment of posterior dentition is justified due to their documented influence on temporomandibular joint (TMJ) structure and function. Posterior tooth loss and reduced occlusal support have been associated with significant alterations in TMJ biomechanics, including changes in condylar position, increased joint loading, and higher stress distribution, which may contribute to the development or progression of temporomandibular disorders (TMD) [25]. Furthermore, edentulism and loss of posterior occlusal contacts can disrupt the stomatognathic system, leading to functional and structural adaptations in the TMJ, including remodeling of joint components and altered joint space relationships [26]. Systematic evidence also suggests that the absence of posterior teeth is associated with an increased risk of TMD due to impaired mastication, occlusal instability, and compensatory neuromuscular activity [27]. Similarly, postoperative trauma to the mandible may directly affect TMJ morphology and function through structural damage, altered loading patterns, or postsurgical remodeling processes, thereby acting as a confounding factor in imaging-based assessments. Additionally, teeth that have undergone endodontic treatment may present altered proprioception and occlusal dynamics, potentially influencing masticatory function and indirectly affecting TMJ biomechanics. Therefore, excluding these conditions is essential to minimize confounding variables and to ensure a more accurate evaluation of TMJ morphological characteristics.

Moreover, the exclusion criteria adopted in this study may have introduced a degree of selection bias. Specifically, CBCT scans showing bone fixation plates suggestive of prior mandibular trauma, as well as individuals without posterior teeth or with all posterior teeth endodontically treated, were excluded to avoid potential confounding factors that could influence temporomandibular joint bone morphology. However, these conditions may be associated with altered functional loading and structural changes in the TMJ. Their exclusion could therefore limit the representativeness of the sample and may have led to an underestimation or overestimation of the true prevalence of bone changes. Consequently, the findings should be interpreted with caution, particularly when extrapolating to broader or more clinically diverse populations. Another limitation of this study is that image evaluation was performed by a single undergraduate examiner. Although prior calibration was conducted, the use of a single observer may introduce variability and potential observer bias. The absence of inter-examiner reliability assessment limits the ability to ensure reproducibility and consistency of the findings. Future studies should consider involving multiple calibrated examiners and reporting inter- and intra-examiner agreement to strengthen methodological robustness.

The high prevalence of TMJ alterations (91.5%) found in this study must be interpreted within the context of clinical significance versus incidental findings. Since clinical symptoms were not assessed, it is essential to consider that morphological changes, particularly flattening (76.4%), often represent a physiological effort of the joint to maintain homeostasis through bone remodeling. As previously reported, such findings may be prevalent even in asymptomatic individuals, functioning as an adaptation to functional mechanical loads rather than a definitive marker of disease [11,14]. This aspect further complicates comparisons with studies that include symptomatic populations, in which the prevalence and type of alterations may reflect pathological processes rather than adaptive remodeling. Clinically, this reinforces that CBCT findings must be strictly correlated with patient symptoms to avoid overdiagnosis of physiological bone remodeling.

The null hypothesis was partially rejected since no relationship was observed between gender and the presence of TMJ alterations (*p* > 0.05), which corroborates with other studies [28,29]. However, it should be noted that discrepancies in the literature regarding gender differences may be influenced by variations in sample composition, including age range, hormonal status, and inclusion of symptomatic patients. Studies focusing on clinical populations or specific age groups, such as postmenopausal women, may report different outcomes compared to imaging-based retrospective analyses like the present study. While some literature suggests a higher female predisposition to TMJ changes [23,30], often attributed to low estrogen levels during menopause, which can detrimentally affect articular cartilage and promote degeneration [31]—this study found no such sexual dimorphism. This lack of significant difference (91.1% in females and 92.4% in males) suggests that, in this specific population, local mechanical factors or individual adaptive capacities may play a more dominant role in condylar bone morphology than systemic gender-related variables [11,14]. Clinically, these findings emphasize that imaging results should be interpreted as potential adaptive responses to functional demands rather than strictly gender-linked pathologies.

Regarding TMJ bone changes and age, in this study, there was a statistical difference between the age of the patients and the presence of osteophytes, sclerosis, erosion and subchondral cysts. However, the magnitude of these age-related differences should be carefully analyzed. For instance, while the prevalence of osteophytes showed a substantial absolute increase of 25 percentage points between the youngest group (43.9% in patients <20 years) and the oldest group (68.9% in patients ≥60 years), other alterations showed more modest trends. Sclerosis affected 24.4% of younger patients compared to 44.4% of older patients, while erosion effectively doubled in prevalence from 12.2% to 24.4%. This study has some limitations that should be considered when interpreting the findings. Due to its cross-sectional design, the present investigation is inherently limited to the assessment of prevalence and associations, precluding any inference of causality between the observed bone changes and potential contributing factors. Therefore, the identified relationships should be interpreted with caution, as temporal sequences and cause–effect mechanisms cannot be established. Longitudinal studies are warranted to better elucidate the progression and clinical significance of these temporomandibular joint alterations.

A relevant aspect that should be considered when interpreting CBCT findings is the potential for overdiagnosis of adaptive bone changes, particularly flattening. Flattening of the mandibular condyle is frequently described as a morphological alteration; however, it may represent a physiological adaptive response to functional loading rather than a pathological condition. The high sensitivity of CBCT allows detection of subtle cortical changes that might not be clinically significant, increasing the likelihood of classifying normal remodeling processes as degenerative alterations. In the absence of clinical correlation, such imaging findings may lead to overestimation of disease prevalence and unnecessary clinical concern. Therefore, caution is warranted when interpreting flattening and similar features, emphasizing the importance of integrating imaging results with clinical signs and symptoms to avoid misdiagnosis and overtreatment. Massila and Sivasubramanian (2016) [31] observed that erosion was the predominant finding in 56.6% of cases, followed in descending order by narrowing of the joint space in 40% of cases, sclerosis in 30%, osteophytes in 16.6% and subchondral cysts in 13.3% of cases. Importantly, this study differs from the present investigation in terms of sample characteristics and possibly diagnostic criteria, which may explain the higher prevalence of erosion reported, highlighting the need for cautious comparison between studies. Elderly patients have been described by other researchers reporting statistically significant differences between the mean age of patients without erosion (46 years) and the mean age of those with moderate erosion (52 years) [23]. No subchondral cysts were observed in patients under the age of 20, but 6.7% were found in the 60+ age group. These findings suggest that remodeling and degenerative changes were more frequent in older age groups, reflecting the cumulative effect of functional load over time [11]. Nevertheless, the fact that a significant portion of the elderly population remains free of certain degenerative signs like erosion (75.6%) highlights the modest effect size of age alone on joint pathology.

## 5. Conclusions

Based on the study model adopted, the most frequently found alteration was flattening, followed by osteophyte, sclerosis, erosion and subchondral cyst. The presence or absence of alterations was not affected by the sex of the individuals analyzed. Although associations were observed between the affected side and age with the presence of TMJ alterations, these findings should be interpreted with caution, as the cross-sectional design does not allow causal inferences and the observed relationships may reflect sample-specific characteristics. Therefore, the results should be considered primarily descriptive, and their clinical applicability remains limited without correlation with functional and symptomatic data.

## Figures and Tables

**Table 1:** Frequency of bone changes in the temporomandibular joint (TMJ) regarding to the gender of the patient.

		Female N (%)	Male N (%)	Total N (%)
Alteration in TMJ	Absent	29 (8.9%)	12 (7.6%)	41 (8.4%)
	Present	297 (91.1%)	145 (92.4%)	442 (91.5%)
Flattening	Absent	74 (22.7%)	40 (25.5%)	114 (23.6%)
	Present	252 (77.3%)	117 (74.5%)	369 (76.4%)
Osteophyte	Absent	152 (46.6%)	75 (47.8%)	227 (47%)
	Present	174 (53.4%)	82 (52.2%)	256 (53%)
Sclerosis	Absent	215 (66%)	112 (71.3%)	327 (67.7%)
	Present	111 (34%)	45 (28.7%)	156 (32.3%)
Erosion	Absent	261 (80.1%)	125 (79.6%)	386 (79.9%)
	Present	65 (19.9%)	32 (20.4%)	97 (20.1%)
Subchondral cyst	Absent	317 (97.2%)	153 (97.5%)	470 (97.3%)
	Present	9 (2.8%)	4 (2.5%)	13 (2.7%)

**Table 2:** Distribution of patients regarding the age and presence of temporomandibular joint (TMJ) alterations.

		Age			
		<20 N (%)	21–39 N (%)	40–59 N (%)	≥60 N (%)
Alteration in TMJ	Absent	3 (7.3%)	25 (10.1%)	11 (7.4%)	2 (4.4%)
	Present	38 (92.7%)	223 (89.9%)	138 (92.6%)	43 (95.6%)
Flattening	Absent	9 (22%)	55 (22.2%)	42 (28.2%)	8 (17.8%)
	Present	32 (78%)	193 (77.8%)	107 (71.8%)	37 (82.2%)
Osteophyte *	Absent	23 (56.1%)	119 (48%)	71 (47.7%)	14 (31.1%)
	Present	18 (43.9%)	129 (52%)	78 (52.3%)	31 (68.9%)
Sclerosis *	Absent	31 (75.6%)	173 (69.8%)	98 (65.8%)	25 (55.6%)
	Present	10 (24.4%)	75 (30.2%)	51 (34.2%)	20 (44.4%)
Erosion *	Absent	36 (87.8%)	204 (82.3%)	112 (75.2%)	34 (75.6%)
	Present	5 (12.2%)	44 (17.7%)	37 (24.8%)	11 (24.4%)
Subchondral cyst *	Absent	41 (100%)	244 (98.4%)	143 (96%)	42 (93.3%)
	Present	0	4 (1.6%)	6 (4%)	3 (6.7%)

* Indicates statistical difference (Pearson’s chi-square test; *p* < 0.05).

**Table 3:** Distribution of the temporomandibular joint (TMJ) alterations in relation to the affected side.

		Left Side N (%)	Right Side N (%)
Alteration in TMJ	Absent	89 (18.4%)	113 (23.4%)
	Present	394 (81.6%)	370 (76.6%)
Flattening	Absent	182 (37.7%)	211 (43.7%)
	Present	301 (62.3%)	272 (56.3%)
Osteophyte	Absent	295 (61.1%)	307 (63.6%)
	Present	188 (38.9%)	176 (36.4%)
Sclerosis	Absent	361(74.7%)	398 (82.4%)
	Present	122 (25.3%)	85 (17.6%)
Erosion	Absent	424(87.8%)	427 (88.4%)
	Present	59 (12.2%)	56 (11.6%)
Subchondral cyst	Absent	477(98.8%)	476 (98.6%)
	Present	6 (1.2%)	7 (1.4%)

## Data Availability

The data that support the findings of this study are available on request from the corresponding author.
